# Supplementary figures and images for: Quantitative UV-C dose validation with photochromic indicators for informed N95 emergency decontamination
Source: PLoS One. 2021 Jan 6;16(1):e0243554. doi: 10.1371/journal.pone.0243554 (PMC7787392; doi:10.1371/journal.pone.0243554)

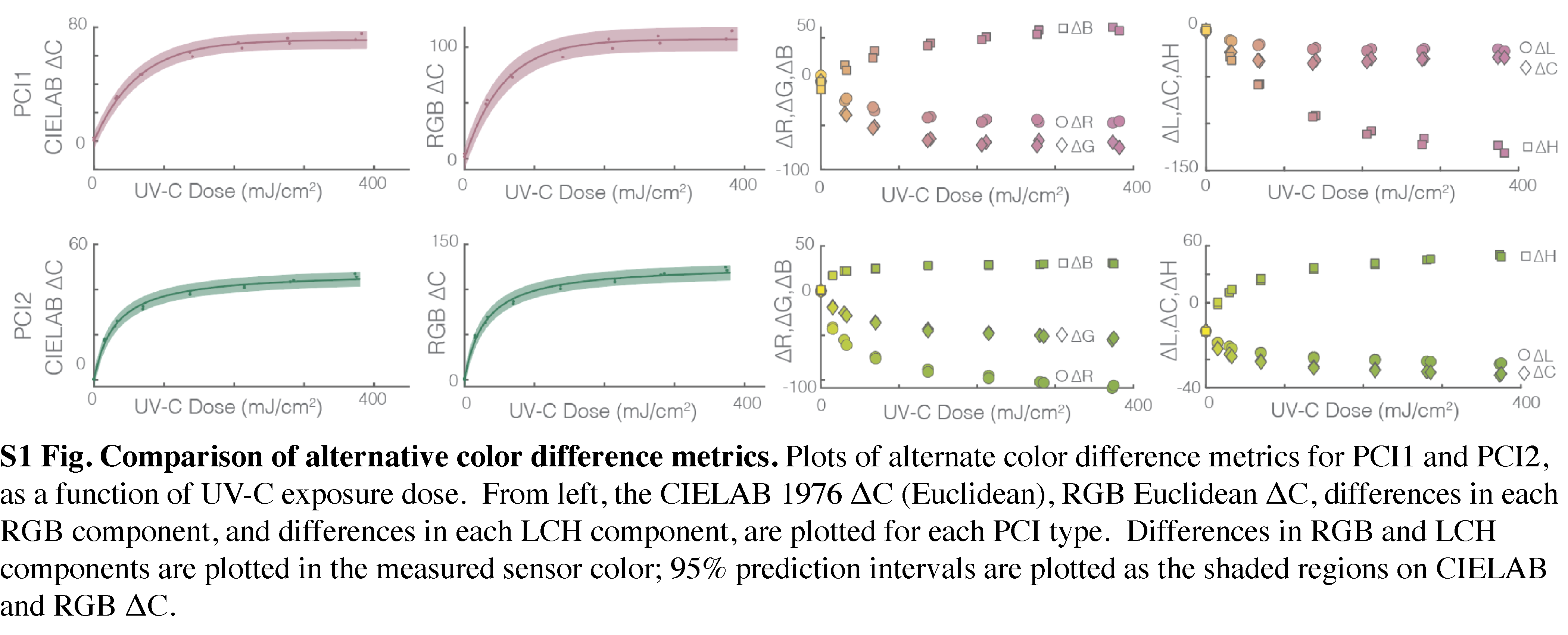

Supplement: S1 Fig — (TIF) [file pone.0243554.s001.tif]

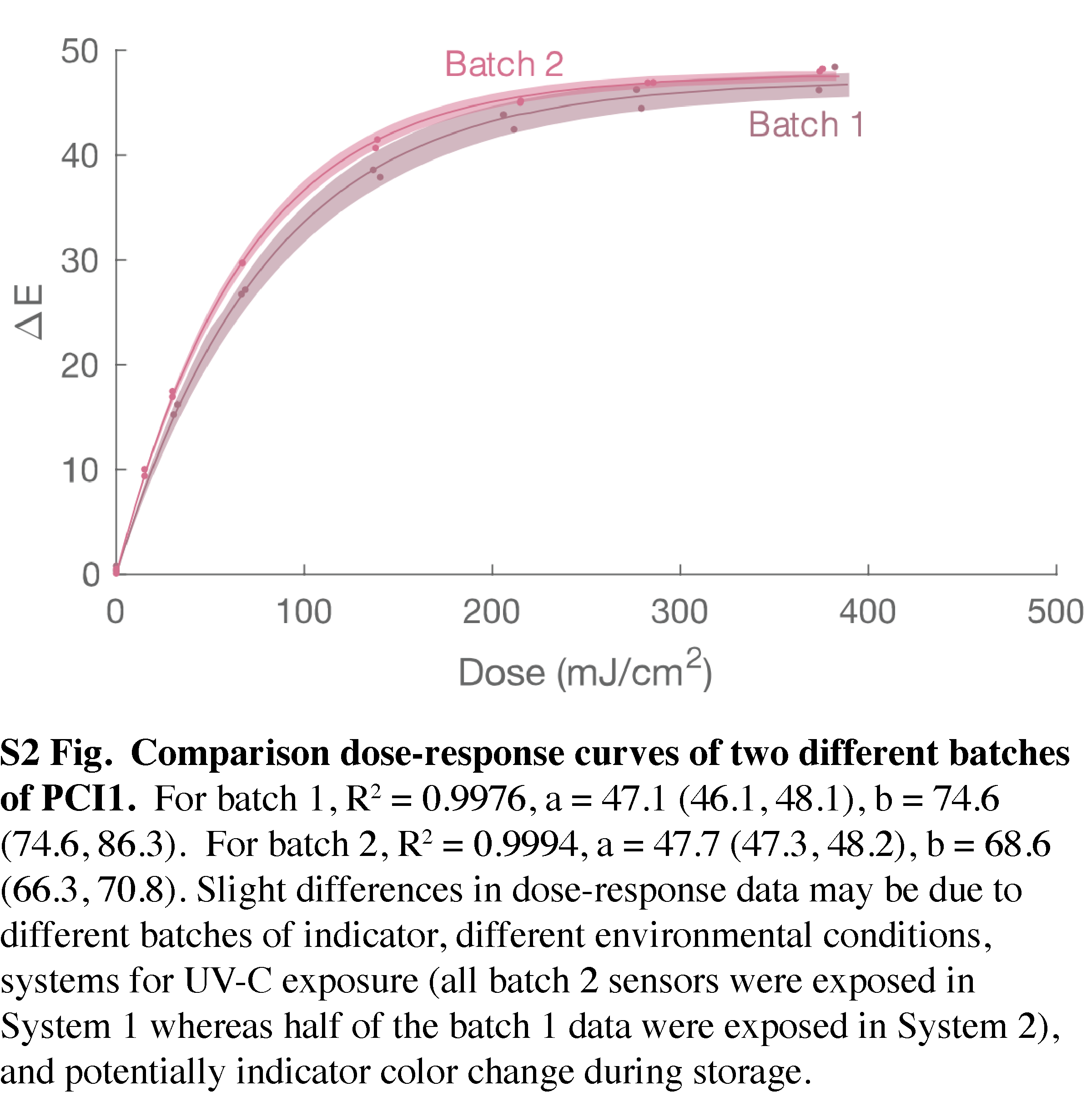

Supplement: S2 Fig — (TIF) [file pone.0243554.s002.tif]

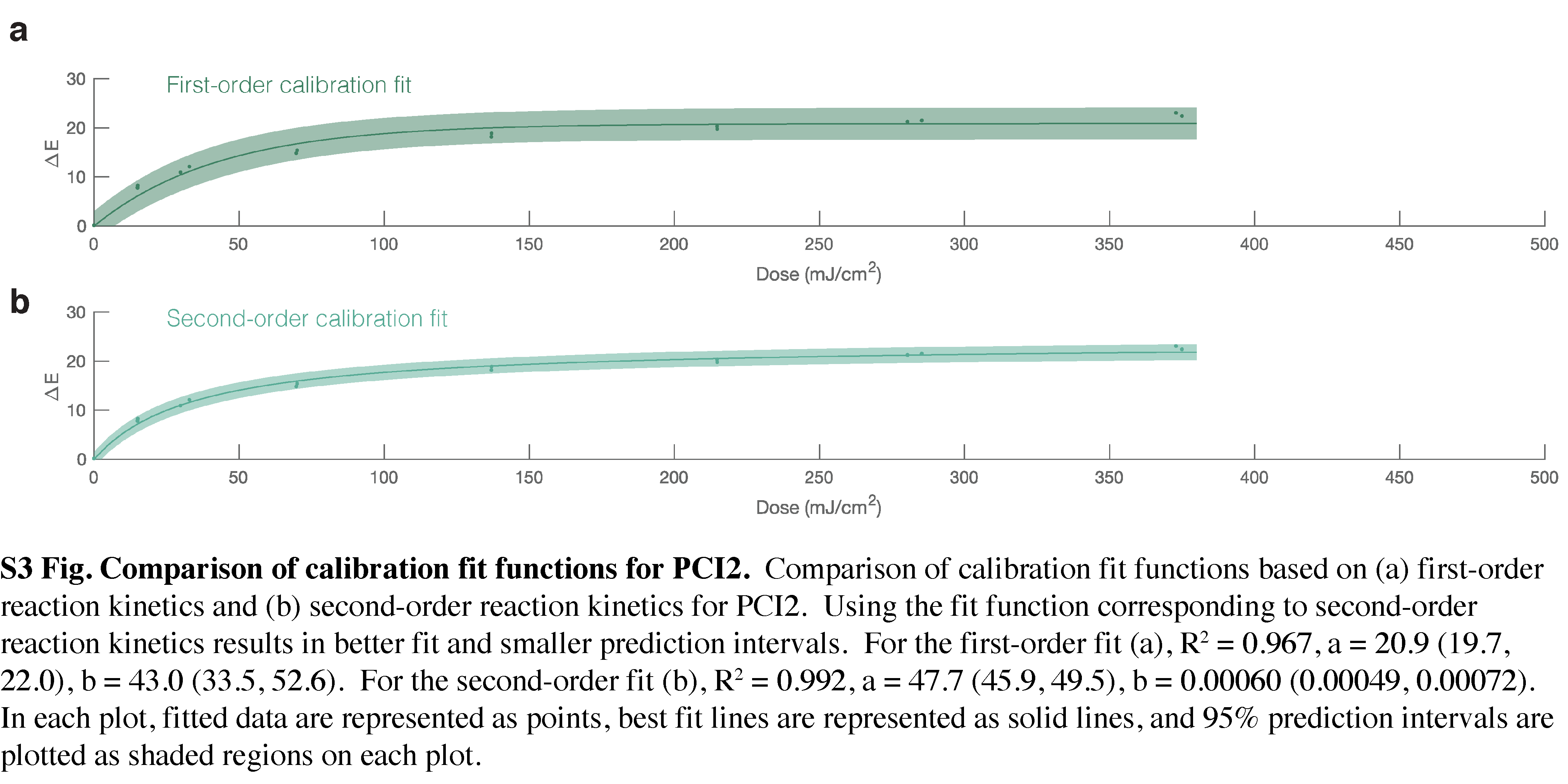

Supplement: S3 Fig — (TIF) [file pone.0243554.s003.tif]

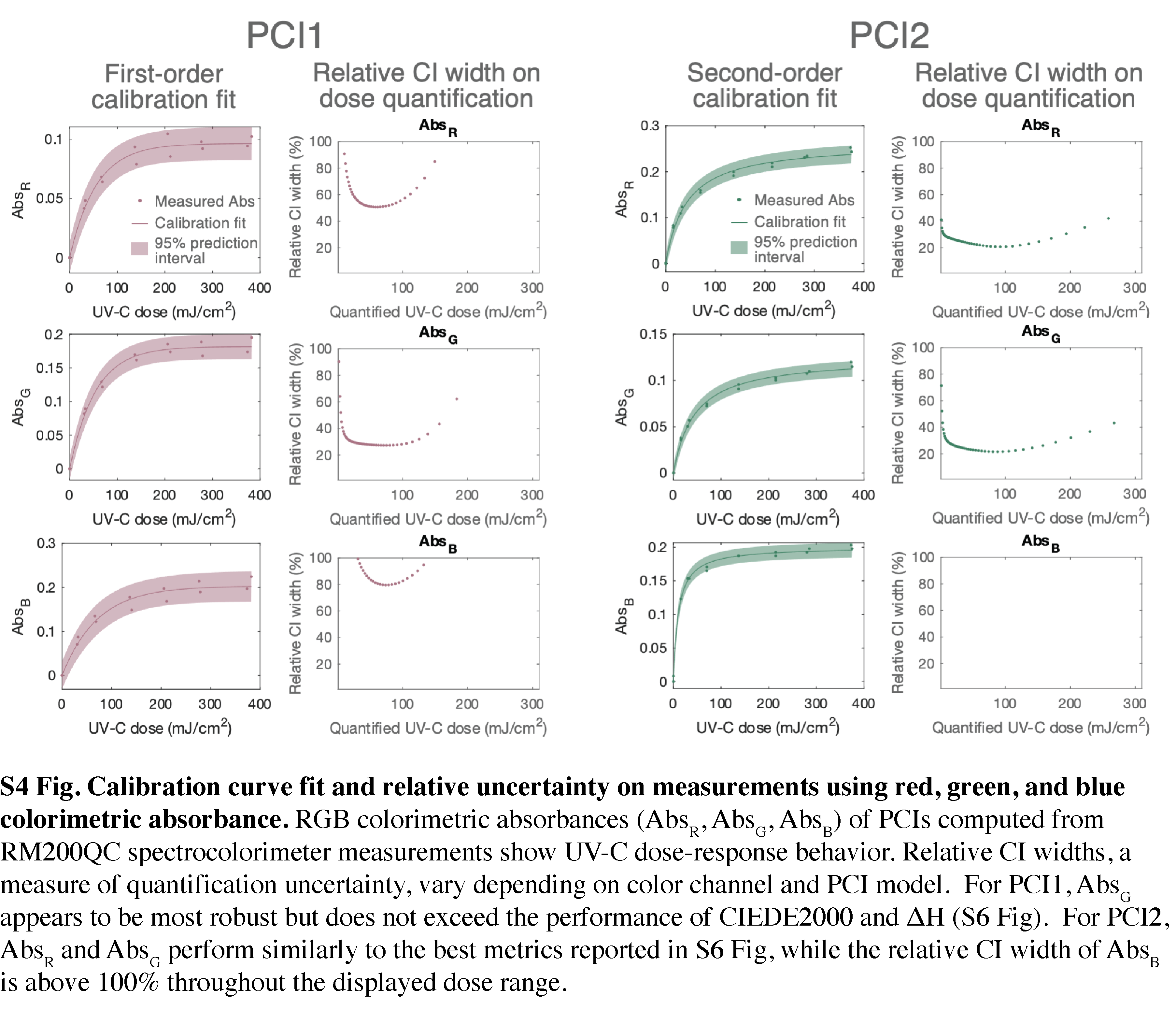

Supplement: S4 Fig — (TIF) [file pone.0243554.s004.tif]

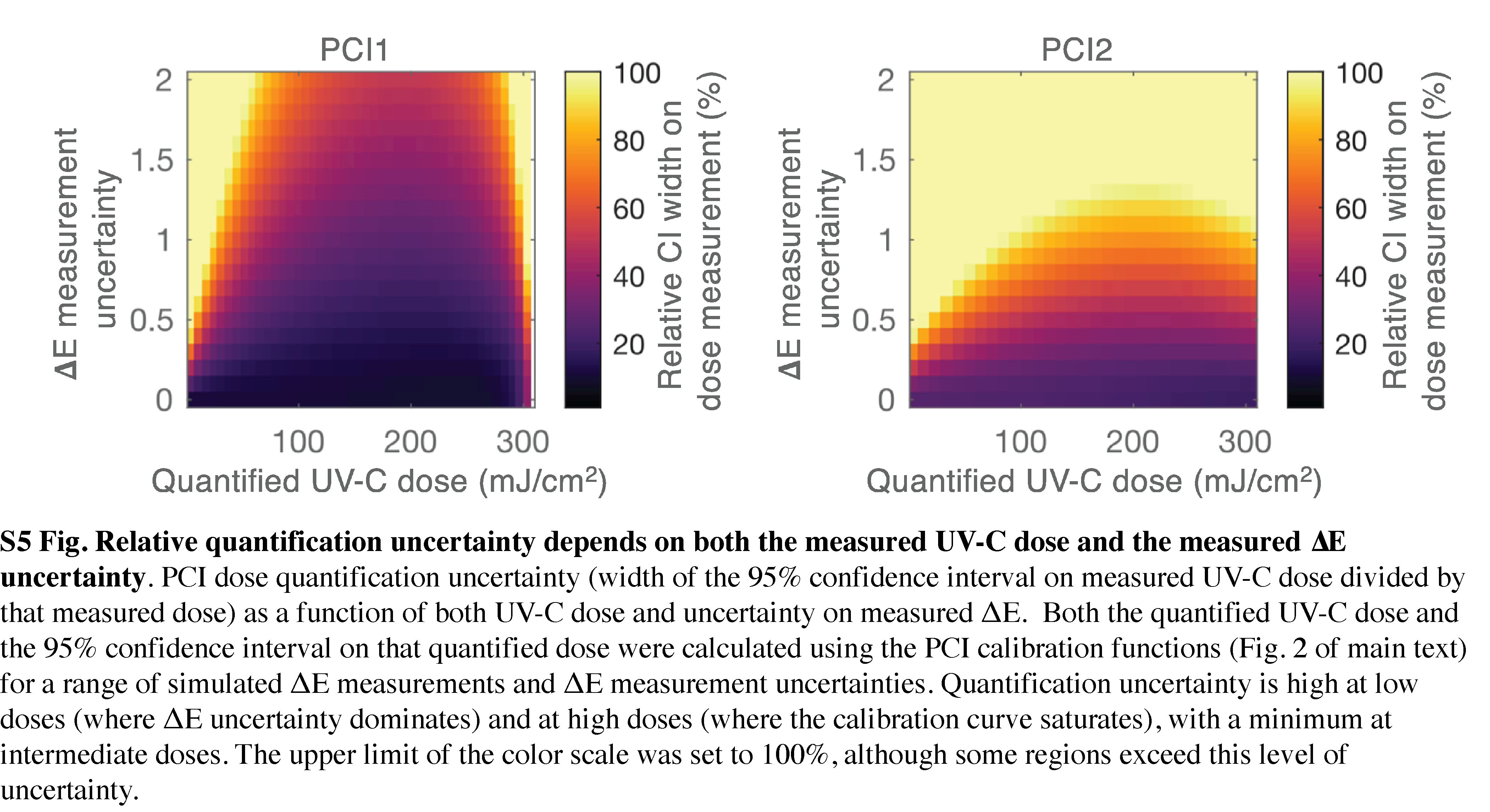

Supplement: S5 Fig — (TIF) [file pone.0243554.s005.tif]

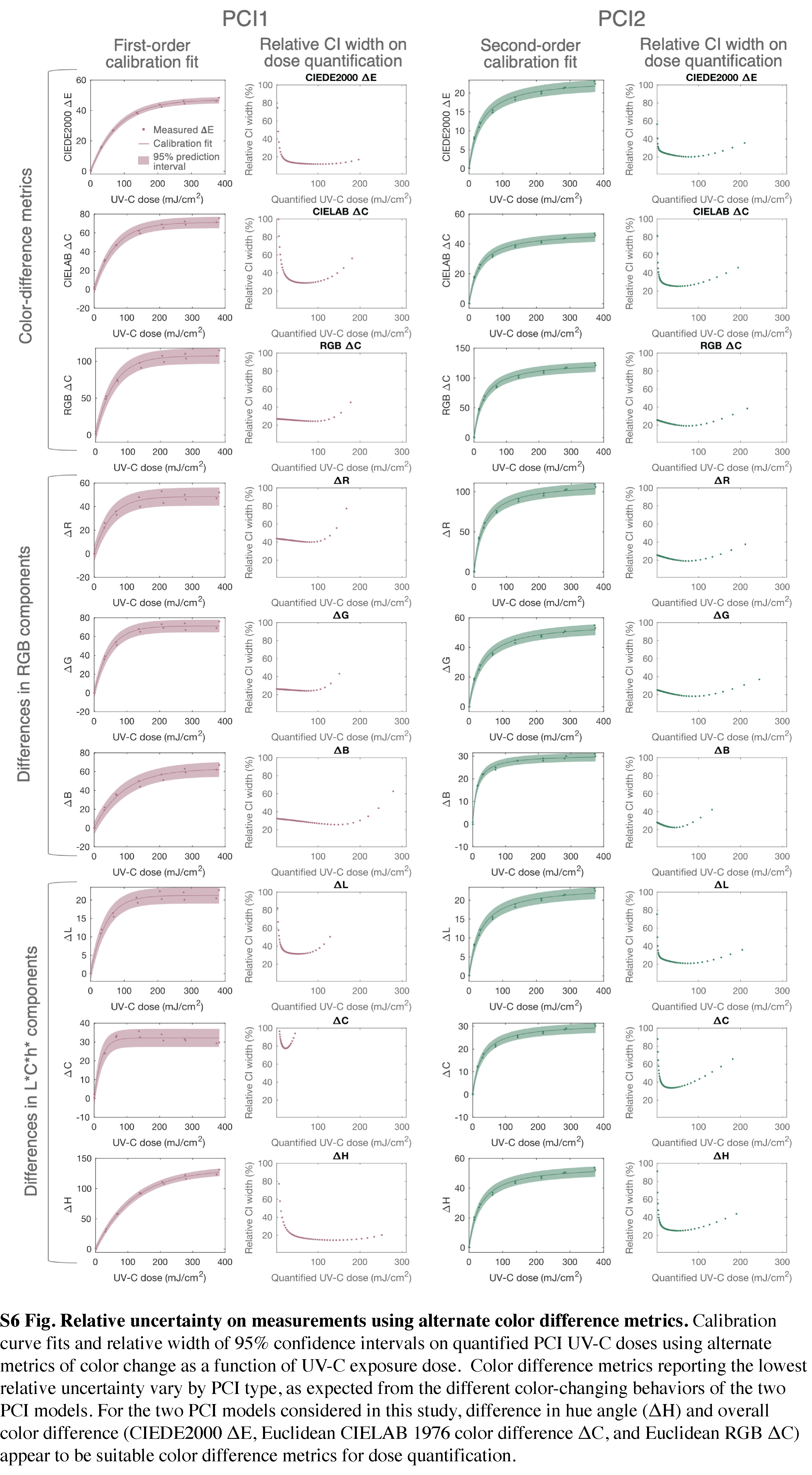

Supplement: S6 Fig — (TIF) [file pone.0243554.s006.tif]

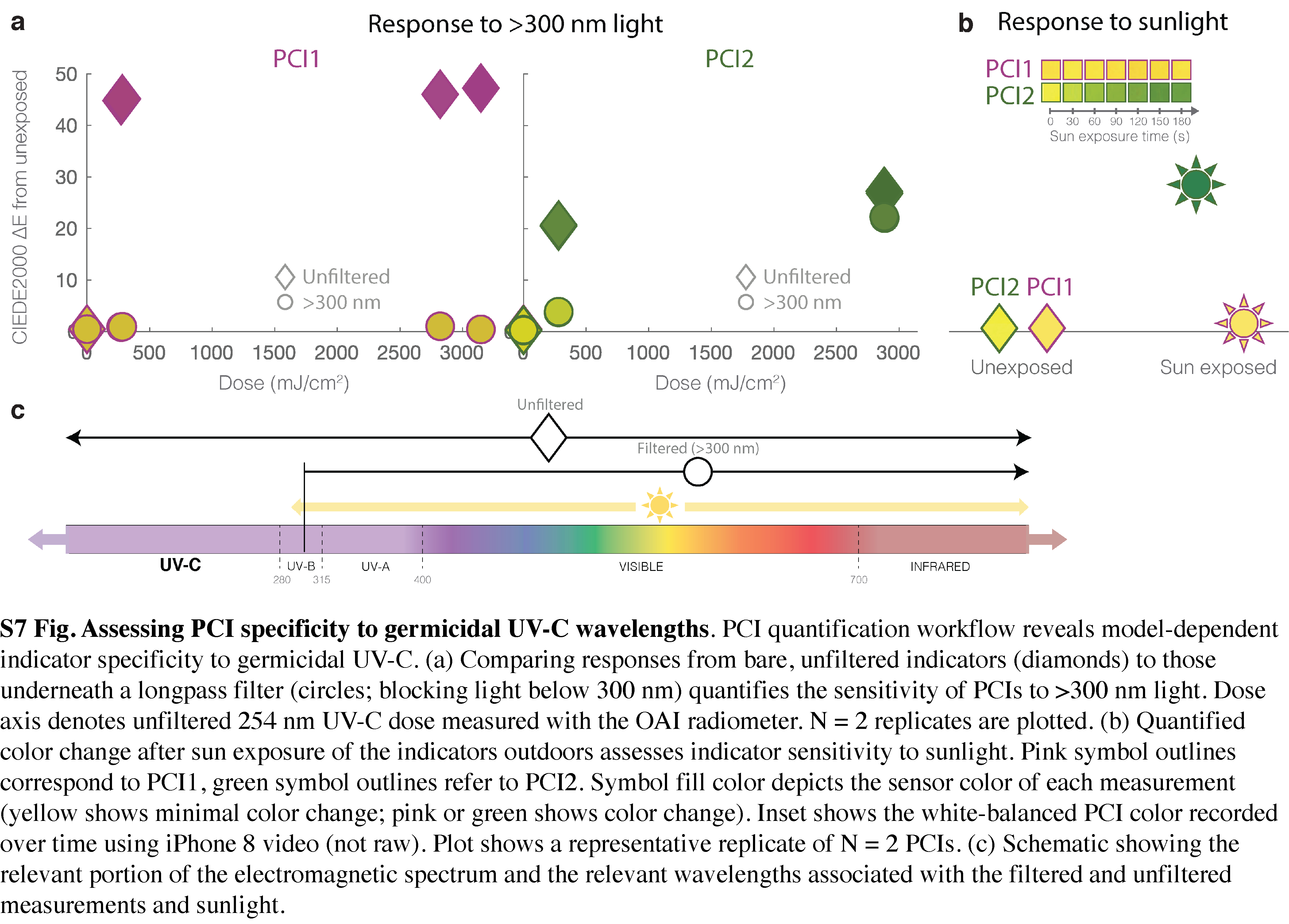

Supplement: S7 Fig — (TIF) [file pone.0243554.s007.tif]

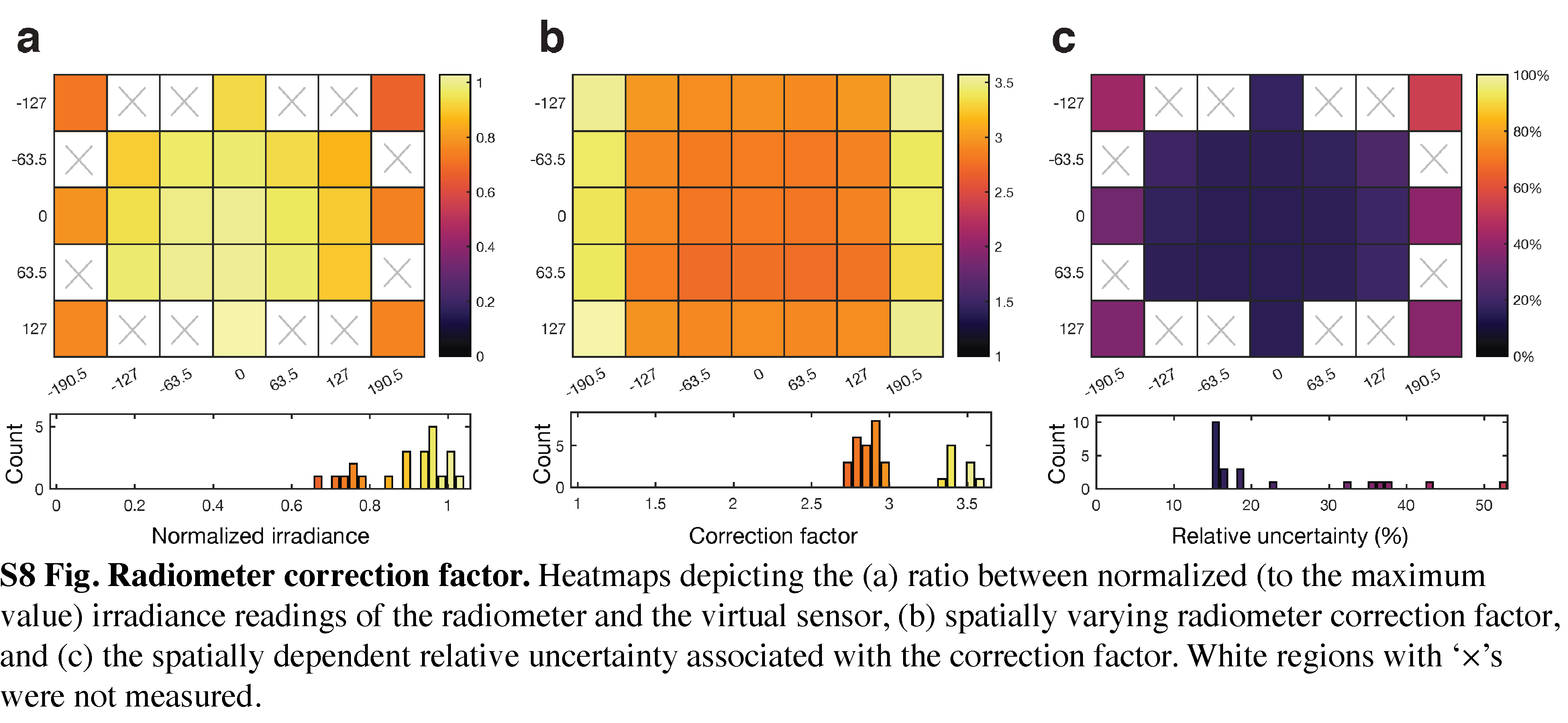

Supplement: S8 Fig — (TIF) [file pone.0243554.s008.tif]

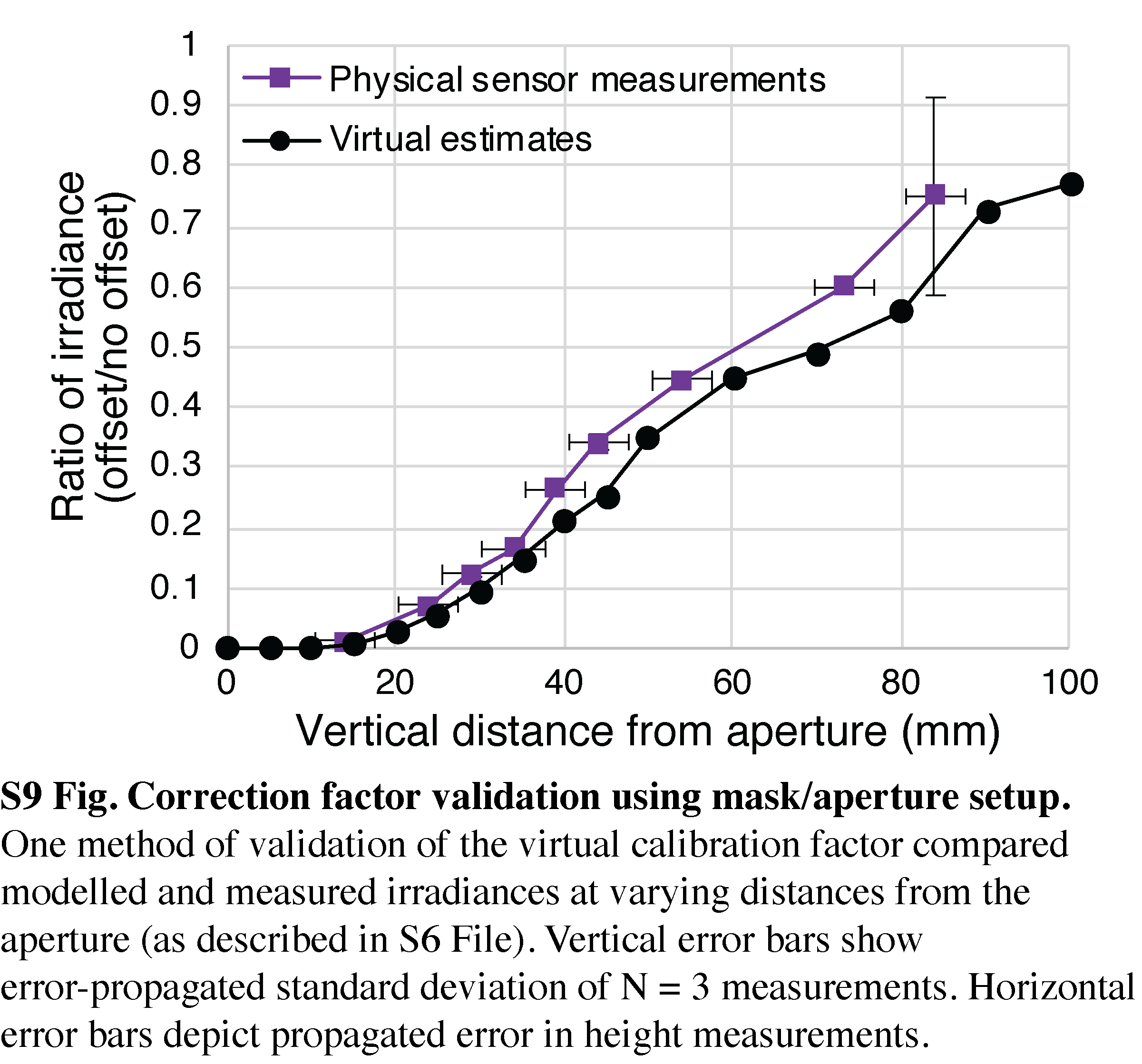

Supplement: S9 Fig — (TIF) [file pone.0243554.s009.tif]

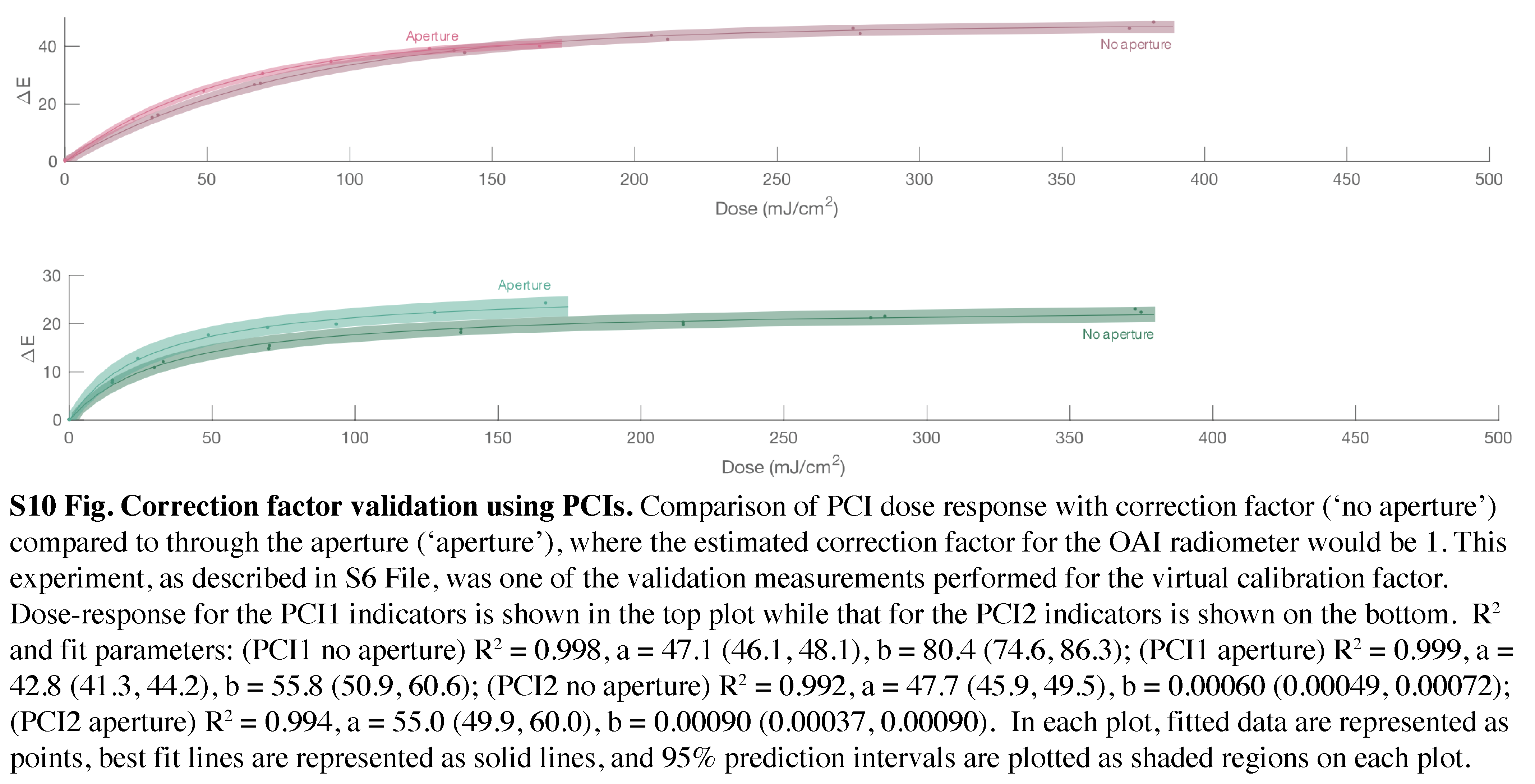

Supplement: S10 Fig — (TIF) [file pone.0243554.s010.tif]

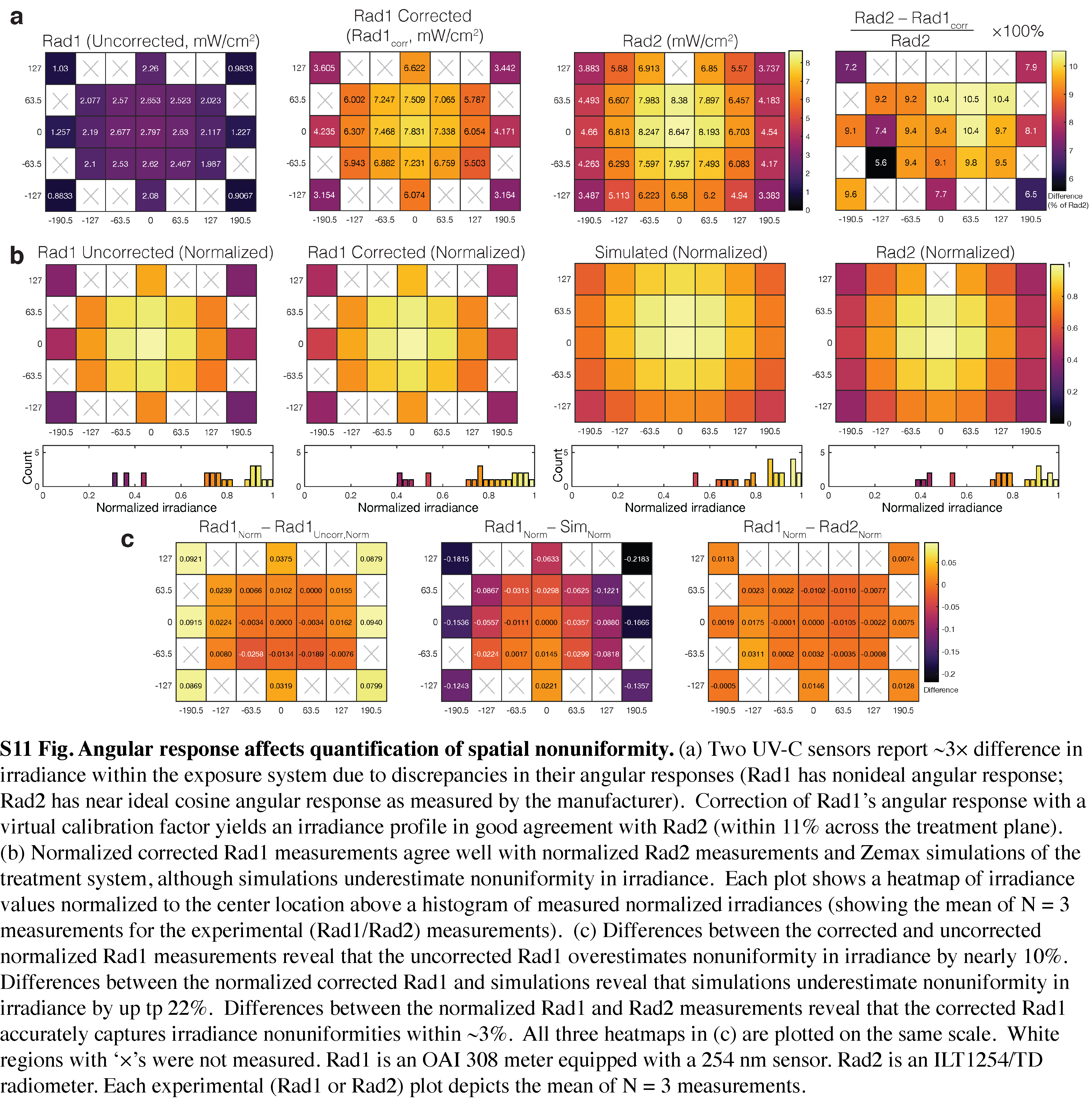

Supplement: S11 Fig — (TIF) [file pone.0243554.s011.tif]

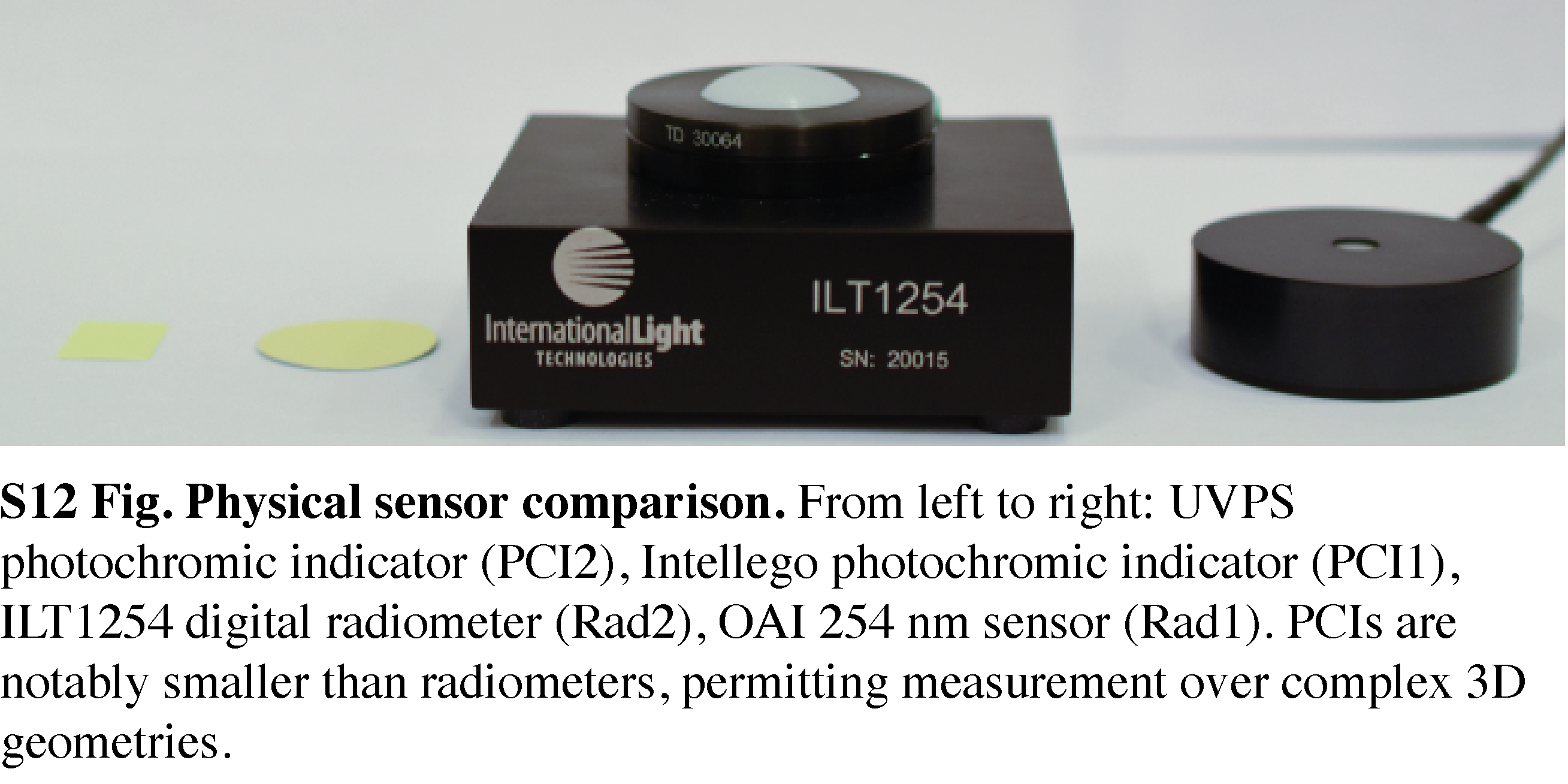

Supplement: S12 Fig — (TIF) [file pone.0243554.s012.tif]

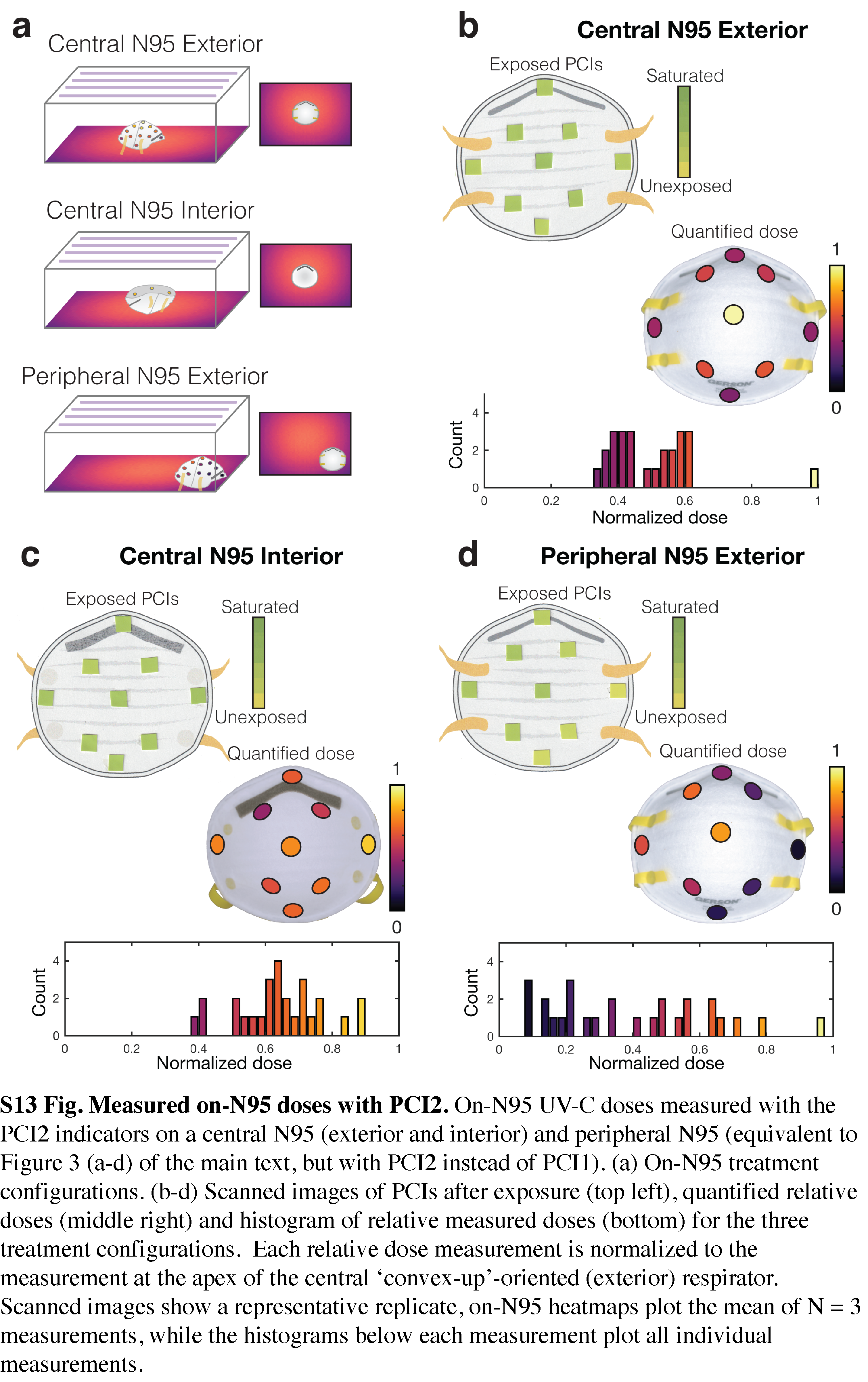

Supplement: S13 Fig — (TIF) [file pone.0243554.s013.tif]

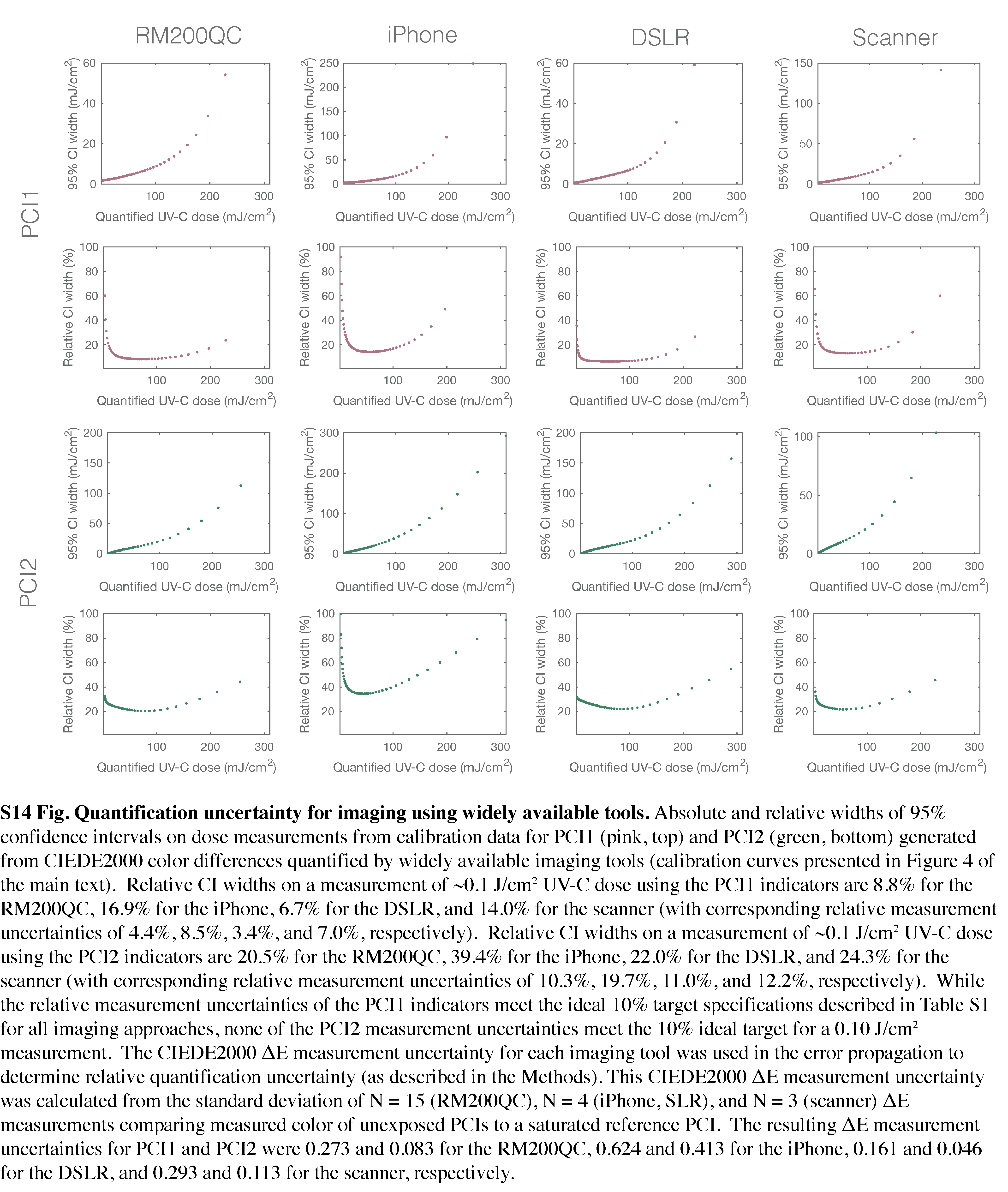

Supplement: S14 Fig — (TIF) [file pone.0243554.s014.tif]

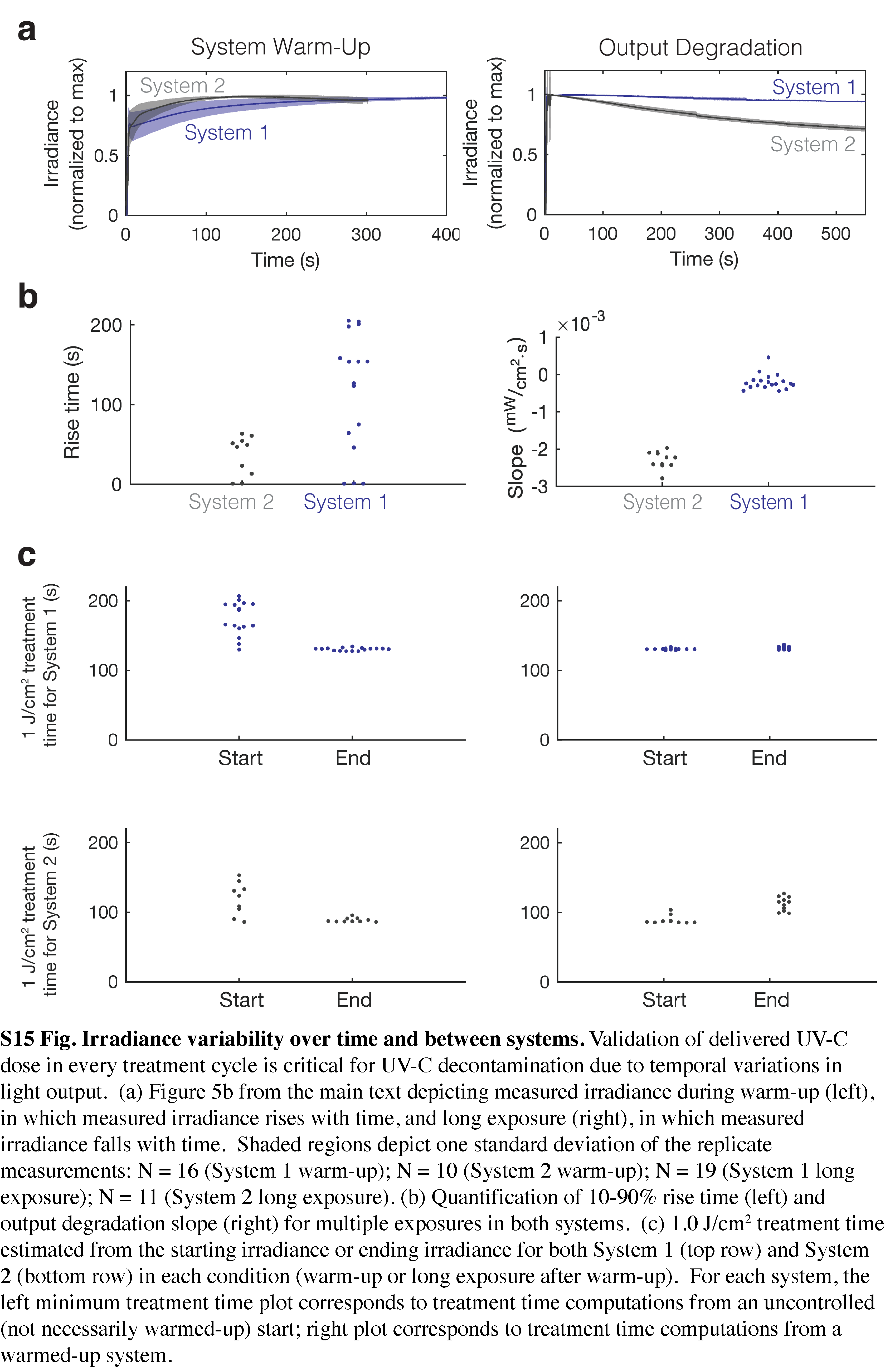

Supplement: S15 Fig — (TIF) [file pone.0243554.s015.tif]

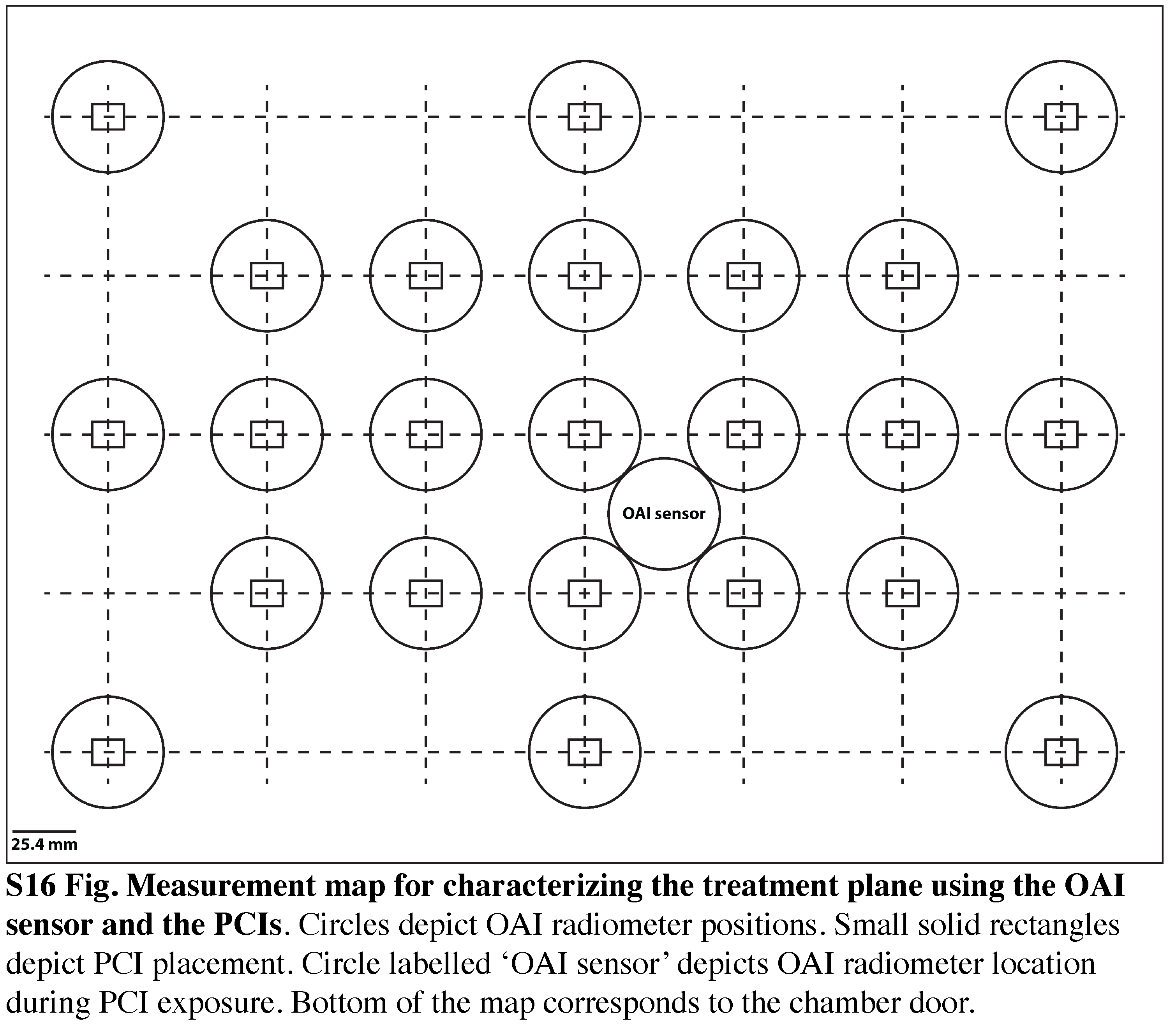

Supplement: S16 Fig — (TIF) [file pone.0243554.s016.tif]

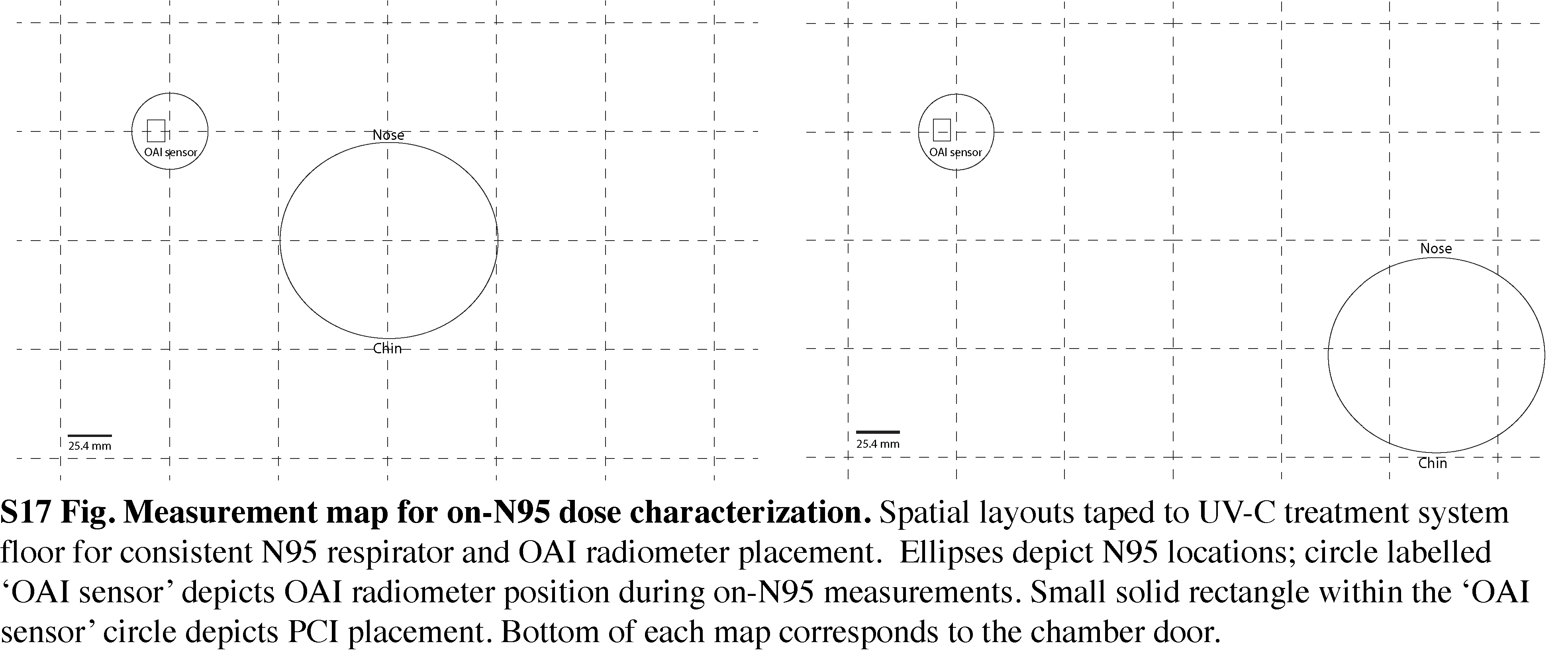

Supplement: S17 Fig — (TIF) [file pone.0243554.s017.tif]

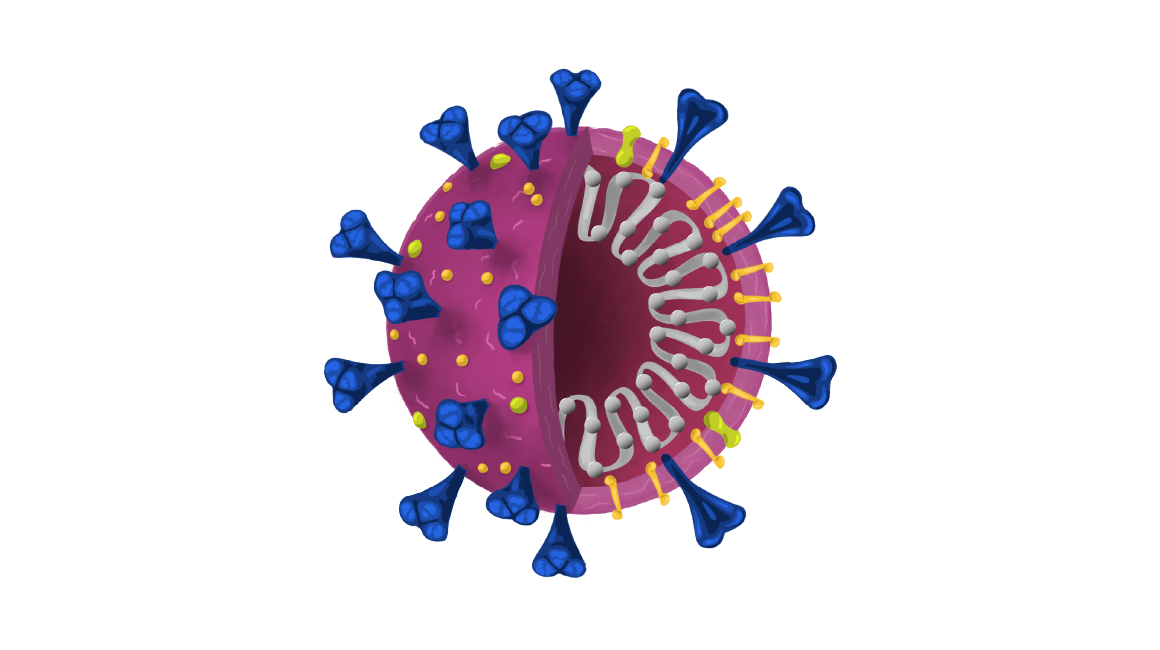

Supplement: S18 Fig — (PNG) [file pone.0243554.s018.png]
