# Supplementary material for: Quantitative UV-C dose validation with photochromic indicators for informed N95 emergency decontamination
Source: PLoS One. 2021 Jan 6;16(1):e0243554. doi: 10.1371/journal.pone.0243554 (PMC7787392; doi:10.1371/journal.pone.0243554)
Supplement: S1 Table — (DOCX) [file pone.0243554.s019.docx]

## **S1 Table.** Specifications for robust UV-C measurements

| **Number** | **Metric** | **Units** | **Marginal value** | **Ideal value** |
| --- | --- | --- | --- | --- |
| 1 | Dose measurement range (in-process validation) | J/cm^2^ | ≥ 1.0 | > 3.0 |
| 2 | Dose measurement range (informed design) | J/cm^2^ | > 0.1 | > 0.3 |
| 3 | Relative uncertainty on dose measurement (CI) | % | < 20 | < 10 |
| 4 | Accuracy | % | > 80 | > 90 |
| 5 | Sensitivity to non-germicidal longer wavelengths | % | < 5 | < 1 |

1. The marginal dose measurement dynamic range for in-process validation (≥1.0 J/cm^2^) is based upon the marginally-acceptable dose to be delivered to each and every N95 surface for ≥3-log inactivation of enveloped viruses (based on peer-reviewed evidence in the scientific literature [1–3]). The measurement method needs to be able to discern whether this dose has been exceeded. Ideally, the measurement range would be higher (>3.0 J/cm^2^) as the location of the reference sensor during decontamination will likely receive a higher dose than the N95 surface receiving the lowest dose due to shadowing and the model-dependent angles of the N95 surfaces.
2. Because informed design of N95 decontamination systems and processes can use relative dose measurements, the necessary dose measurement dynamic range for informed design can be lower than that for in-process validation. >0.1 J/cm^2^ was chosen as the marginal value for this application to ensure that the UV-C exposure times for informed design were no less than 1/10^th^ those for in-process validation. As informed design uses the same exact UV-C exposure system as that used for the actual decontamination process, low dynamic range PCIs would require very short exposure times because the systems are designed to deliver ≥1.0 J/cm^2^ during a reasonable exposure time. These short exposure times during informed design may (1) not be feasible or (2) introduce unacceptable degrees of run-to-run variability.
3. The calibration uncertainty for very well characterized UV-C radiometers is ~5% [4] (although many radiometers will not reach this level due to sources of error in UV-C measurements [5]). As measurement solutions like photochromic indicators (PCIs) have advantages over even the best calibrated radiometers (e.g., form factor), we identified a marginal target for PCIs of 4 times the value for radiometers, and an ideal target as twice the radiometer value. These values (20% and 10%, respectively) allow reasonable ‘safety factors’ of <50% to be implemented to ensure minimally-acceptable doses are reached. Safety factors are multipliers on the target dose to take into account uncertainty on the measurements (e.g., for 20% total propagated uncertainty, one may want to use a safety factor of 1.5 and ensure at least ≥1.5 J/cm^2^ was delivered to all N95 surfaces)
4. Accuracy values (how well measurements align with a calibrated, NIST-traceable reference measurement) were chosen to align with target relative uncertainty.
5. Ideally, UV-C measurements for decontamination characterization and validation should only report irradiance or dose within the germicidal range (UV-C extends to 280 nm; germicidal efficacy at 300 nm is <10% of that at 254 nm [6]). We selected marginal and ideal values such that the measurement response to >300 nm was 1-2 orders of magnitude less than that to <300 nm light from a commonly employed low-pressure mercury/amalgam source.

References:

1. Heimbuch B, Harnish D. Research to Mitigate a Shortage of Respiratory Protection Devices During Public Health Emergencies [Internet]. Applied Research Associate, Inc; 2019 [cited 2020 Mar 27]. Report No.: HHSF223201400158C. Available from: https://www.ara.com/wp-content/uploads/MitigateShortageofRespiratoryProtectionDevices_3.pdf

2. Lore MB, Heimbuch BK, Brown TL, Wander JD, Hinrichs SH. Effectiveness of Three Decontamination Treatments against Influenza Virus Applied to Filtering Facepiece Respirators. Ann Occup Hyg. 2011 Aug 22;56(1):92–101.

3. Heimbuch BK, Wallace WH, Kinney K, Lumley AE, Wu C-Y, Woo M-H, et al. A pandemic influenza preparedness study: Use of energetic methods to decontaminate filtering facepiece respirators contaminated with H1N1 aerosols and droplets. Am J Infect Control. 2011 Feb;39(1):e1–9.

4. Larason T, Ohno Y. Calibration and characterization of UV sensors for water disinfection. Metrologia. 2006 Apr;43(2):S151–6.

5. Larason TC, Cromer CL. Sources of error in UV radiation measurements. J Res Natl Inst Stand Technol. 2001 Jul;106(4):649.

6. Lytle CD, Sagripanti J-L. Predicted Inactivation of Viruses of Relevance to Biodefense by Solar Radiation. J Virol. 2005 Nov 15;79(22):14244–52.
