# Supplementary material for: Quantitative UV-C dose validation with photochromic indicators for informed N95 emergency decontamination
Source: PLoS One. 2021 Jan 6;16(1):e0243554. doi: 10.1371/journal.pone.0243554 (PMC7787392; doi:10.1371/journal.pone.0243554)
Supplement: S2 Table — (DOCX) [file pone.0243554.s020.docx]

S2 Table. Quantified on-N95 relative doses (normalized to the apex location 1 of the exterior of the central respirator) measured with PCIs as depicted in S13 Fig as well as Fig 3 of the main text. Errors are reported as one standard deviation propagated uncertainty encompassing both the standard deviation of 3 replicate measurements (measured across ≥ 2 separate days) and the uncertainty on the dose measurement from the calibration curve. Schematics in the left column depict measurement location for each configuration.

| **Configuration** | **Location** | **Norm. Dose (PCI1)** | **Norm. Dose (PCI2)** |
| --- | --- | --- | --- |
| Central N95 Exterior 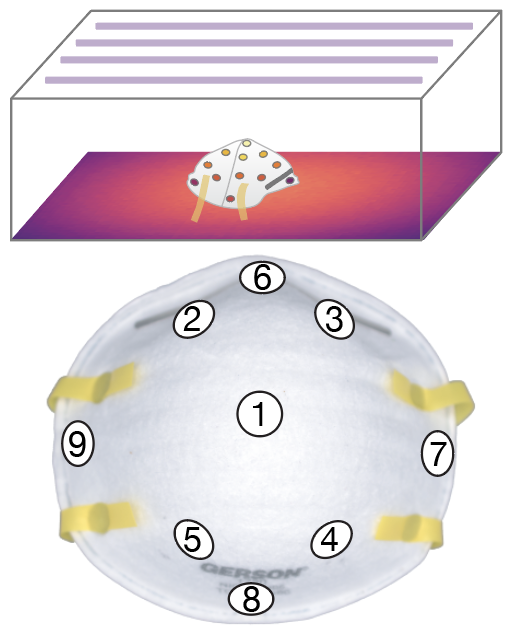 | 1 | 1.00 ± 0.07 | 1.00 ± 0.07 |
|  | 2 | 0.47 ± 0.06 | 0.56 ± 0.04 |
|  | 3 | 0.38 ± 0.05 | 0.51 ± 0.05 |
|  | 4 | 0.53 ± 0.07 | 0.61 ± 0.04 |
|  | 5 | 0.57 ± 0.06 | 0.59 ± 0.04 |
|  | 6 | 0.36 ± 0.02 | 0.43 ± 0.04 |
|  | 7 | 0.33 ± 0.02 | 0.39 ± 0.03 |
|  | 8 | 0.28 ± 0.04 | 0.35 ± 0.03 |
|  | 9 | 0.38 ± 0.03 | 0.43 ± 0.04 |
| Central N95 Interior 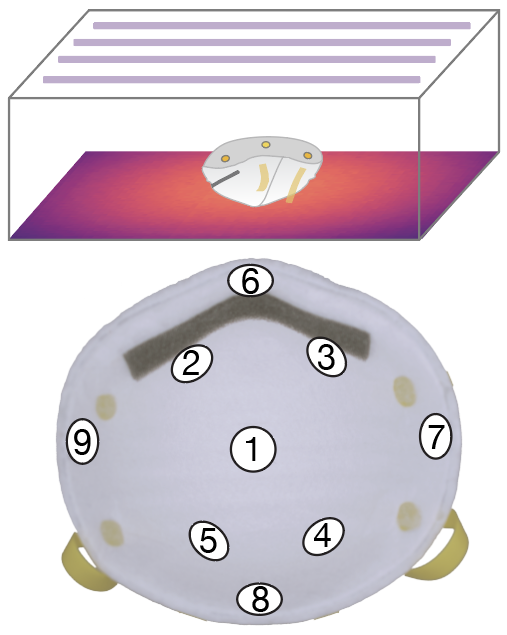 | 1 | 0.75 ± 0.06 | 0.74 ± 0.06 |
|  | 2 | 0.40 ± 0.03 | 0.40 ± 0.03 |
|  | 3 | 0.48 ± 0.04 | 0.52 ± 0.04 |
|  | 4 | 0.66 ± 0.06 | 0.68 ± 0.06 |
|  | 5 | 0.61 ± 0.05 | 0.59 ± 0.05 |
|  | 6 | 0.60 ± 0.06 | 0.63 ± 0.05 |
|  | 7 | 0.90 ± 0.06 | 0.87 ± 0.07 |
|  | 8 | 0.62 ± 0.04 | 0.65 ± 0.05 |
|  | 9 | 0.73 ± 0.06 | 0.73 ± 0.07 |
| Peripheral N95 Exterior 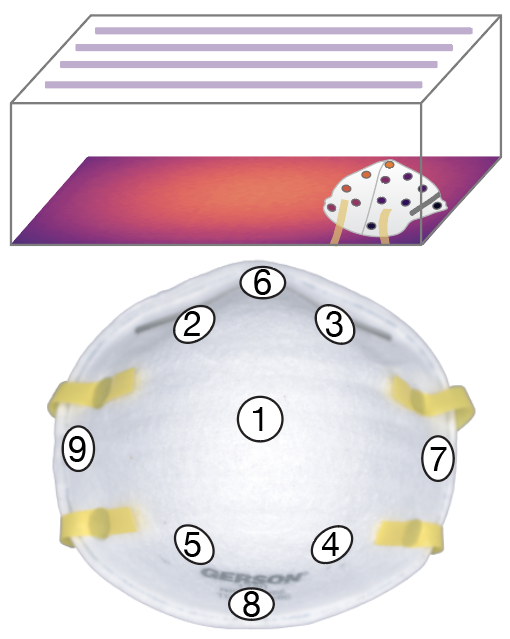 | 1 | 0.71 ± 0.07 | 0.78 ± 0.16 |
|  | 2 | 0.65 ± 0.07 | 0.66 ± 0.11 |
|  | 3 | 0.24 ± 0.03 | 0.25 ± 0.05 |
|  | 4 | 0.16 ± 0.02 | 0.207 ± 0.019 |
|  | 5 | 0.43 ± 0.06 | 0.47 ± 0.04 |
|  | 6 | 0.34 ± 0.04 | 0.37 ± 0.05 |
|  | 7 | 0.063 ± 0.011 | 0.081 ± 0.008 |
|  | 8 | 0.104 ± 0.015 | 0.143 ± 0.014 |
|  | 9 | 0.55 ± 0.06 | 0.58 ± 0.07 |
