# Supplementary material for: Quantitative UV-C dose validation with photochromic indicators for informed N95 emergency decontamination
Source: PLoS One. 2021 Jan 6;16(1):e0243554. doi: 10.1371/journal.pone.0243554 (PMC7787392; doi:10.1371/journal.pone.0243554)
Supplement: S1 File — (DOCX) [file pone.0243554.s021.docx]

## **S1 File: Informed UV-C system design and validation PCI workflow example**

As depicted in Fig 1(d) of the main text, in this work we introduce a novel PCI-based dose quantification workflow for UV-C N95 decontamination systems. Step 1 of the workflow involves quantification and calibration of the PCIs. Steps 2-3 involve informed design of UV-C treatment. Step 4 involves in-process validation of every treatment cycle.

**Step 1**: The first step of the workflow is to create a robust calibration curve of PCI color change as a function of dose, using a calibrated, NIST-traceable (or traceable to similar standards organization) radiometer (Fig 1(d, i)). The tested doses should cover the full range of the PCI with sufficient data points to yield a well-defined calibration curve fit (we typically acquired data at 7-10 doses), with replicate indicators run at each dose. Color measurements of the PCIs exposed to each dose, along with unexposed and saturated reference indicators, should be acquired immediately (PCI color is often unstable after exposure) with a color measurement tool like a spectrocolorimeter or spectrophotometer. A more widely available imaging tool can also be used as long as (1) acquisition parameters are tightly controlled and held constant, (2) raw images are acquired, with the exposed PCI and reference within the same image, (3) the PCIs are isolated from ambient illumination, (4) images are not saturated, and (5) the ΔE measurement uncertainty has been quantified. Color differences between each PCI and the unexposed reference, as a function of measured UV-C dose, are calculated and fitted to a calibration curve as described in the Methods and S3 File. This calibration curve can then be applied to estimate UV-C dose from PCI color change in future experiments. Calibration should be confirmed after changes in condition (e.g., temperature, humidity) and after change in PCI shipment/lot. Example calibration curves are presented in Fig 2 and Fig 4 of the main text.

**Step 2**: After the calibration curve is generated, relative exposures can be mapped across the UV-C treatment plane using either a single exposure of spatially arrayed PCIs or multiple exposures of a radiometer moved to each position in order to select the best representative N95 and reference positions for on-N95 measurements (Fig 1(d, ii)). To make these measurements, PCIs are placed in defined regions of the treatment plane and exposed to a quantifiable UV-C dose (as determined from the calibration curve of Step 1). UV-C dose at each location is estimated from the calibration curve based on the color difference between each exposed PCI and an unexposed reference PCI using the same acquisition criteria as described in Step 1. Variability around the treatment plane as well as the lowest- and highest-dose regions should be identified at this step, as demonstrated in Fig 2 of the main text. The active area of the treatment plane (area where N95s will ultimately be placed), as well as a reference location within the treatment plane (low-dose region that will not be shadowed by N95s during treatment), should be defined in this step. The inclusion of the reference facilitates translation from informed design to in-process validation in Step 4, when dose may be monitored using only a PCI at the reference location.

**Step 3**: 3D structure adds additional and significant variability to the UV-C dose delivered to N95 surfaces (Fig 1(d, iii)). Steep or shadowed surfaces will receive the lowest dose on a given object, so these regions should be identified on each model of N95 to be decontaminated. PCIs should be placed on these regions as well as regions expected to receive high doses (e.g., N95 apex). Note that the 3D structure, and therefore optimal PCI placement, is expected to be model-dependent (e.g., some models may have pleats that cause shadowing while others may have steeper morphology). To assess the full degree of variability in irradiance to which N95 surfaces may be exposed, representative N95s with PCIs affixed in the expected high and low-dose regions should be placed in the active regions of the treatment plane expected to have (1) the highest dose, and (2) the lowest dose (informed from Step 2). A PCI placed at the reference location during on-N95 measurement enables relative doses delivered to all N95 surfaces to be compared to those at the reference location. PCIs at all locations should be exposed to a UV-C dose within the indicator’s quantifiable range. Using the color quantification workflow and calibration curve, relative doses delivered to N95 surfaces in the high and low-dose regions of the treatment plane can then be computed with respect to the dose at the reference location. From this relative dose information, the configuration of N95s within the treatment plane can be optimized (to minimize on-N95 dose variation). Any adjustments to the N95 layout should be reanalyzed to determine the relative dose quantification in the adjusted setup. The minimum dose required at the reference location to ensure all N95 surfaces receive ≥1.0 J/cm^2^ should also be assessed. For example, if the lowest-dose N95 region receives 25% of the dose delivered to the reference location, the reference indicator must receive ≥4.0 J/cm^2^ in order for all N95 surfaces to receive at least the marginally effective dose of 1.0 J/cm^2^. This reference position and minimum dose can then be used for in-process validation of UV-C treatment. An example of on-N95 dose measurement is presented in Fig 3 of the main text.

**Step 4**: After identifying the reference indicator location and minimum dose the reference must receive to ensure all surfaces of the N95s receive ≥1.0 J/cm, an indicator with sufficient dynamic range should be placed at that reference location during each and every UV-C treatment cycle to validate that the minimum reference dose is delivered to the reference location. To extend PCI dynamic range, optical attenuation can be coupled with PCIs (to reduce light reaching the PCI by a known factor, stretching the dynamic range of response to include the minimum reference dose). Examples of optical attenuation are presented in Fig 5 of the main text.
