# Supplementary material for: Quantitative UV-C dose validation with photochromic indicators for informed N95 emergency decontamination
Source: PLoS One. 2021 Jan 6;16(1):e0243554. doi: 10.1371/journal.pone.0243554 (PMC7787392; doi:10.1371/journal.pone.0243554)
Supplement: S2 File — (DOCX) [file pone.0243554.s022.docx]

## **S2 File: Comparison of color difference metrics outlined in ASTM D2244-16**

Seeking to compare several of the additional metrics of color difference outlined in ASTM D2244-16, we computed and plotted several additional metrics as a function of UV-C dose (S1 Fig) [1]. The RGB and CIELAB $\Delta C$, as well as the CIE 1976 Metric Hue Difference ($\Delta H$), showed strong dose-dependent response. These dose-dependent relationships suggest that any of these established metrics could be employed for PCI calibration. A previous study characterized an unspecified model of UV Process Supply PCI and defined a color difference metric as $\Delta R+\Delta G$; these previous characterization data appeared to have higher variability than our measurements but with qualitatively similar saturation [2]. Our more robust quantification is likely due to (1) our use of a dedicated, contact color measurement tool instead of a camera [3,4] and (2) our direct, logged measurements of UV-C dose to generate the calibration curve rather than modelled irradiance. As the CIEDE2000 metric [5] showed comparable dose-response to the other metrics and has been found to better correlate with perceptible color difference and outperform other color difference standards [6,7], we chose to employ CIEDE2000 $\Delta E$ as the color difference metric for subsequent calibration and measurement in our characterization and application of the PCIs.

References:

1. ASTM International. Standard Practice for Calculation of Color Tolerances and Color Differences from Instrumentally Measured Color Coordinates [Internet]. ASTM D2244 - 16. 2016 [cited 2020 Dec 3]. Available from: https://www.astm.org/Standards/D2244.htm

2. Solari F, Girolimetti G, Montanari R, Vignali G. A New Method for the Validation of Ultraviolet Reactors by Means of Photochromic Materials. Food Bioprocess Technol. 2015 Nov;8(11):2192–211.

3. Cui G, Luo MR, Rhodes PA, Rigg B, Dakin J. Grading textile fastness. Part 1; Using a digital camera system. Color Technol. 2003 Jul;119(4):212–8.

4. Nixon M, Outlaw F, MacDonald LW, Leung TS. The importance of a device specific calibration for smartphone colorimetry. Color Imaging Conf. 2019 Oct 21;2019(1):49–54.

5. Luo MR, Cui G, Rigg B. The development of the CIE 2000 colour-difference formula: CIEDE2000. Color Res Appl. 2001 Oct;26(5):340–50.

6. Gómez-Polo C, Muñoz MP, Lorenzo Luengo MC, Vicente P, Galindo P, Martín Casado AM. Comparison of the CIELab and CIEDE2000 color difference formulas. J Prosthet Dent. 2016 Jan;115(1):65–70.

7. Luo MR. CIEDE2000, History, Use, and Performance. In: Luo MR, editor. Encyclopedia of Color Science and Technology [Internet]. New York, NY: Springer New York; 2016. p. 202–7. Available from: https://doi.org/10.1007/978-1-4419-8071-7_7
