# Supplementary material for: Quantitative UV-C dose validation with photochromic indicators for informed N95 emergency decontamination
Source: PLoS One. 2021 Jan 6;16(1):e0243554. doi: 10.1371/journal.pone.0243554 (PMC7787392; doi:10.1371/journal.pone.0243554)
Supplement: S3 File — (DOCX) [file pone.0243554.s023.docx]

## **S3 File: Generation of PCI calibration functions**

We endeavored to define a calibration function mapping the color change of photochromic UV-C indicators to quantitative UV-C dose (fluence). For some types of UV indicator [1], the sensing mechanism involves one molecule (acid-release agent, ARAH) that is triggered by UV light to release a proton (H^+^), which then protonates another dye molecule (D^–^), resulting in a color change:

$$ARAH\to{ARA}^{-}+H^{+}$$

$$D^{-}+H^{+}\to DH$$

To design an appropriate calibration function to which to fit color change data (CIEDE2000 [2] color differences from an unexposed sensor) as a function of UV dose (*dose*), we hypothesized that, depending on the rate-limiting step of the reaction, one might be able to use functions based on expected product concentration from first- or second-order reaction kinetics. For first-order reaction kinetics [3]:

$$A\underset{\to}{k_{1}}B$$

$$\left[ B \right]={[A]}_{0}\left\{ 1-e^{-k_{1}t} \right\}$$

Assuming the color change is proportional to the concentration of product, and using the relationship that $dose=irradiance\cdot t$:

$$\Delta E\approx a\left\{ 1-e^{-\frac{dose}{b}} \right\}$$

Where $b\equiv irradiance/k_{1}$. We found that this first-order kinetics fit function fit the PCI1 color change data well. However, we noted that the fit was poorer for PCI2, with lower goodness-of-fit and poor visual agreement (as depicted in S3 Fig). We hypothesized that a fit function derived from second-order reaction kinetics might yield better fit performance for the PCI2 model. As a first pass, we chose the simplest type of second-order reaction [3]:

$$A+A\underset{\to}{k_{2}}B$$

$$\frac{1}{\left[ A \right]}=\frac{1}{{[A]}_{0}}+k_{2}t$$

$$\left[ A \right]=\frac{{[A]}_{0}}{1+{[A]}_{0}k_{2}t}$$

$$\left[ B \right]=\frac{\left[ A \right]_{0}-\left[ A \right]}{2}=\frac{\left[ A \right]_{0}\left\{ 1-\frac{1}{1+\left[ A \right]_{0}k_{2}t} \right\}}{2}=\frac{\frac{1}{2}{[A]}_{0}^{2}{\cdot k}_{2}t}{1+\left[ A \right]_{0}k_{2}t}$$

$$\Delta E\approx\frac{\frac{1}{2}a^{2}\cdot b\cdot dose}{1+a\cdot b\cdot dose}$$

This function based on second-order reaction kinetics better fits the PCI2 data, as depicted in S3 Fig.

Due to the two-stage reaction described by Mills, *et al.* [1], it may be more relevant to use an equation derived from consecutive first-order reactions [3]:

$$A\underset{\to}{k_{1}}B\underset{\to}{k_{2}}C$$

$$\left[ C \right]={[A]}_{0}\left\{ 1+\frac{1}{k_{1}-k_{2}}\left[ k_{2}e^{-k_{1}t}-k_{1}e^{-k_{2}t} \right] \right\}$$

$$\Delta E\approx c\left\{ 1+\frac{1}{a-b}\left[ be^{-a\cdot dose}-ae^{-b\cdot dose} \right] \right\}$$

However, when this equation was applied to data from PCI1, the fitting algorithm could not robustly quantify all three fit parameters (with 10 points fitted and R^2^=0.9993; the 95% CI on fit parameter *b* extended from -1.3E+08 to 1.3E+08). This was likely because fit parameter *a* (0.001398) was found to be much smaller than fit parameter *b* (2328), suggesting that the color change is limited by one of the constituent reactions (and thus may be approximated as a single first-order reaction). Similarly, when the fit for a consecutive first-order reaction was applied to PCI2, the second fit parameter was again poorly defined and the goodness-of-fit similar to that for the equation based on a single first-order reaction. We note that although these fit functions serve as effective calibration functions with high goodness-of-fit, the current implementation does not facilitate extraction of reaction parameters (e.g., reaction order, reaction rate) from the curve fit because the relationship between CIEDE2000 and colored reaction product concentration is not known.

After fitting the calibration function to the CIEDE2000 vs. UV-C dose data, 95% prediction intervals on the fit were generated using the MATLAB^®^ ‘predint’ function, generating non-simultaneous observation bounds. The upper bound of this prediction interval, with the addition of a safety factor, could be used to generate a color change threshold to determine whether a given UV-C dose (e.g., 1.0 J/cm^2^) has been surpassed.

References:

1. Mills A, McDiarmid K, McFarlane M, Grosshans P. Flagging up sunburn: a printable, multicomponent, UV-indicator that warns of the approach of erythema. Chem Commun. 2009;(11):1345–6.

2. Luo MR, Cui G, Rigg B. The development of the CIE 2000 colour-difference formula: CIEDE2000. Color Res Appl. 2001 Oct;26(5):340–50.

3. El Seoud OA, Baader WJ, Bastos EL. Practical Chemical Kinetics in Solution. In: Wang Z, editor. Encyclopedia of Physical Organic Chemistry, 5 Volume Set [Internet]. Hoboken, NJ, USA: John Wiley & Sons, Inc.; 2016 [cited 2020 May 29]. p. 1–68. Available from: http://doi.wiley.com/10.1002/9781118468586.epoc1012
