# Supplementary material for: Quantitative UV-C dose validation with photochromic indicators for informed N95 emergency decontamination
Source: PLoS One. 2021 Jan 6;16(1):e0243554. doi: 10.1371/journal.pone.0243554 (PMC7787392; doi:10.1371/journal.pone.0243554)
Supplement: S4 File — (DOCX) [file pone.0243554.s024.docx]

## **S4 File: Assessing specificity of PCIs to germicidal UV-C**

Given that ultraviolet decontamination efficacy depends on wavelength, it is critical that PCIs used for UV-C decontamination are not only sensitive, but also specific to germicidal UV-C wavelengths (200-280 nm)[1]. Although low-pressure mercury sources are often denoted as ‘monochromatic’ at 254 nm, they emit several additional peaks in the UV-B-to-visible range [2] that could lead to overestimation of UV-C dose without UV-C-specificity. In order to assess the sensitivity of PCIs to longer, non-germicidal wavelengths, we compared the PCI color change after exposure through a longpass (>300 nm) optical filter to that of an unfiltered PCI. Both PCIs were exposed at the same time to an unfiltered dose of 0.28 J/cm^2^ as measured by corrected Rad1. We observed that while PCI1 underwent negligible color change under the longpass filter, PCI2 changed color considerably, with ~19% of the color difference attributable to non-germicidal UV wavelengths (S7(a) Fig). Natural sunlight (which contains UV-A and UV-B, but no longer contains UV-C at the earth’s surface [3]) also induced rapid color change ($\Delta E$ ~28 in 5 minutes) of PCI2, while PCI1 only marginally changed ($\Delta E$ ~1.5) (S7(b) Fig). These results highlight the importance of specificity to UV-C measurement tools, as common UV-C sources (e.g., low and medium pressure mercury bulbs, LEDs) vary in their proportional outputs within the germicidal UV-C range [1]. Misreporting the germicidal dose could have wide-ranging negative consequences, so it is essential to accurately assess dose in the germicidal range.

References:

1. Kowalski W. Ultraviolet Germicidal Irradiation Handbook: UVGI for Air and Surface Disinfection [Internet]. Berlin, Heidelberg: Springer Berlin Heidelberg; 2009 [cited 2020 Apr 17]. Available from: http://link.springer.com/10.1007/978-3-642-01999-9

2. Schmid J, Hoenes K, Rath M, Vatter P, Hessling M. UV-C inactivation of Legionella rubrilucens. GMS Hyg Infect Control. 2017 Apr 10;12:1–6.

3. CDC. Ultraviolet Radiation [Internet]. 2019 [cited 2020 Jun 15]. Available from: https://www.cdc.gov/nceh/radiation/ultraviolet.htm
