# Supplementary material for: Quantitative UV-C dose validation with photochromic indicators for informed N95 emergency decontamination
Source: PLoS One. 2021 Jan 6;16(1):e0243554. doi: 10.1371/journal.pone.0243554 (PMC7787392; doi:10.1371/journal.pone.0243554)
Supplement: S5 File — (DOCX) [file pone.0243554.s025.docx]

## **S5 File: Radiometer correction factor determination**

The OAI radiometer was calibrated by the manufacturer using a collimated source such that the sensor is situated a specified distance from the source, at normal geometry (i.e., perpendicular to the optical axis). In this study, however, the sensor is used to measure light that is uncollimated and at non-normal geometric configuration. Since the sensor assembly consists of a cylindrical case where optical elements such as diffusers, filters, and apertures are stacked in front of the photosensing element, the offset distance from the front aperture to the photosensing element causes positional sensitivity when the sensor is used to measure uncollimated beams. Light rays with a high angle of incidence may be blocked from reaching the sensing element. Therefore, we anticipate that the irradiance reported by the photosensing element may be lower than the actual irradiance at the sensor’s front surface, and correction or calibration needs to be performed to compensate for the sensor’s nonideal (non-cosine) angular response.

The inaccuracy in irradiance measurement can be corrected experimentally by calibrating the sensor in the exact conditions for which it will be used; however, in the absence of a reference sensor with an ideal cosine angular response and a photosensing element at the same height as the inaccurate radiometer’s photosensing element, this is not possible. Furthermore, because this calibration must be conducted within each specific UV-C system (since systems are frequently sold separate from radiometers), this experimental approach is not practical to the majority of users. Instead of performing this calibration experimentally, this sensor ‘calibration’ can be performed virtually with the use of optical modeling software to determine spatially dependent ‘correction factors’ that quantify the amount by which the sensor underestimates true irradiance. The sensor’s irradiance readings can then be multiplied by the appropriate correction factor to more accurately reflect the true irradiance.

Colleagues at NIST modelled our specific UV-C source and sensor in Zemax OpticStudio Pro (Version 19.4 SP2). The UV-C bulbs were simulated using known radiation patterns of T8 bulbs. The radiometer was simulated using known physical characteristics of the actual sensor such as the height of the aperture and the angular response of the photodetector, which were furnished by the manufacturer. Monochromatic spectral bandpass at 254 nm was assumed. Since the angular response was only measured up to an angle of incidence of 55° by the manufacturer, the response to angles >55° was extrapolated using a Gaussian fit in Labview. The virtual sensor was validated by first comparing its angular response to a simulated collimated light beam at different angles of incidence to the normalized angular response provided by the manufacturer of the radiometer, and then comparing the spatially resolved normalized virtual irradiance estimates with those of the physical sensor (S8(a) Fig). As shown in S8(a) Fig, the ratios of the normalized irradiances detected by the virtual and physical sensors are close to one, as expected.

The correction factor is calculated as the ratio of the total irradiance incident upon the front surface of the sensor to the irradiance reported by the physical sensor. The total irradiance from the six bulbs at the plane of the radiometer sensor front surface was mapped across the simulated treatment plane. The virtual sensor was then positioned at various points on that same plane to map what the actual sensor readings would be. The resulting correction factor map is shown in S8(b) Fig. The values of the correction factor vary from 2.72-3.57 and are spatially dependent, with larger correction factors required around the periphery of the treatment plane. The corrected and uncorrected radiometer readings were compared to readings from another radiometer with near-ideal (cosine) angular response, as well as compared to simulation. Absolute irradiance readings between the corrected first radiometer and near-ideal cosine second radiometer agreed within 11% (S11(a) Fig). Furthermore, even though the simulated irradiance map underestimates the extent of nonuniformity present in the treatment plane, the relative nonuniformities measured by the virtually corrected radiometer agreed within 3% with those measured by the radiometer with near-ideal cosine response (S11(c) Fig).

The spatially resolved relative uncertainty was calculated as the square root of the sums of the squared relative uncertainties on (1) the OAI calibration (reported by the manufacturer as 3%), (2) the UV-C treatment system’s irradiance ray trace standard uncertainty (standard deviation of N = 4 ray traces), (3) the solid angle uncertainty, represented as the relative difference between the virtual and physical sensor readings (similar error is depicted in S8(a) Fig), and (4) the detector area. This spatially resolved relative uncertainty is shown in S8(c) Fig. The relative error on the detector area was estimated as the relative error on the differences between the virtual and physical OAI sensor readings during a validation experiment using a mask/aperture setup (described in S6 File) designed such that the small aperture limited the angles of incidence to near-normal.

The reported correction factors are only accurate for the specific conditions assumed for this virtual sensor calibration. Changes to the physical chamber, sensor or bulb properties, as well as changes to the height and/or angle of the sensor within the treatment system require revised simulations that account for these new conditions. In addition, changes to the sensor such as blockage of the effective aperture or insertion of optical elements are significant changes not accounted for in the simulation and require recalculation of the correction factors. However, changes to the lamp irradiance as lamps age or are replaced are expected; sensor readings will reflect such changes and the calculated correction factors should still apply.
