# Supplementary material for: Quantitative UV-C dose validation with photochromic indicators for informed N95 emergency decontamination
Source: PLoS One. 2021 Jan 6;16(1):e0243554. doi: 10.1371/journal.pone.0243554 (PMC7787392; doi:10.1371/journal.pone.0243554)
Supplement: S6 File — (DOCX) [file pone.0243554.s026.docx]

## **S6 File: Correction factor validation using a mask/aperture setup**

To validate the computed correction factors, a mask/aperture setup was used to limit the angular distribution of rays that reach the sensor to verify that the virtual sensor matches the actual sensor in positional sensitivity. The mask/aperture setup used a black cardstock-lined cardboard insert to block all emitted UV-C light within the treatment system except for a precisely positioned 6.35 mm aperture. The virtual calibration was repeated with only one source bulb. A mask containing a 6.35 mm diameter aperture was simulated directly below and centered on the virtual bulb. This mask/aperture setup for the experimental setup was mimicked using cardboard, black cardstock, and an Iris Diaphragm (M-ID-1.0, Newport) with aperture set to ~6.35 mm diameter. All but 2 bulbs were removed from the physical UV-C source (2 bulbs were required for operation). The mask/aperture was arranged in the UV-C treatment system such that the aperture was centered directly below one bulb and the mask bolstered using popsicle sticks kept vertical using sticky tack. In order to minimize contributions of the second bulb to the irradiance measurements, a strip of black cardstock was vertically affixed to the side of the aperture in between the two bulbs. Minimal crosstalk was validated as ≤ 0.01 mW/cm^2^ difference in irradiance measured at equidistant offsets from the sensor position below the aperture perpendicular to the long axis of the bulbs.

The experiment was conducted by measuring the irradiance detected by the physical sensor both directly normal to the aperture and laterally offset (parallel to the long axis of the bulbs) by 25.4 mm at several known heights below the bulb. The ratio of these measurements was compared to those predicted by running the equivalent simulation in Zemax OpticStudio. The results are shown in S9 Fig. To further validate the correction factor, PCIs were exposed in the modified mask/aperture setup (in which the correction factor is approximately 1), and the dose-response curve was compared to the curve generated in the original UV-C setup. The results are shown in S10 Fig. While we do not observe perfect agreement between the dose-responses, some variation is expected due to the drastically different irradiances (~0.10 mW/cm^2^ under the aperture; ~7.5-12 mW/cm^2^ without the aperture) measured in the two setups (PCI response as characterized by the manufacturer has some dependence on irradiance [1]).

Reference:

1. Stefan Källberg. UVC exposure (254 nm) of UV sensitive material at different irradiation levels [Internet]. Borås, Sweden: RISE Research Institutes of Sweden; 2017 Nov [cited 2020 Jun 23]. Report No.: MTt7P06268-3. Available from: http://intellego-technologies.com/wp-content/uploads/2020/05/RISE_report_Intellego_UVCdoimeter_100.pdf
