# Supplementary material for: Quantitative UV-C dose validation with photochromic indicators for informed N95 emergency decontamination
Source: PLoS One. 2021 Jan 6;16(1):e0243554. doi: 10.1371/journal.pone.0243554 (PMC7787392; doi:10.1371/journal.pone.0243554)
Supplement: S7 File — (DOCX) [file pone.0243554.s027.docx]

## **S7 File:** Nonuniformities in and between UV-C sources necessitate rigorous UV-C dose characterization

As irradiance is dependent on myriad factors (e.g., UV-C source and surrounding physical environment, operating temperature, bulb conversion efficiency and warm-up status [1,2]), we hypothesized that irradiance variability within and between UV-C treatment systems would necessitate full in-process (vs. snap-shot) validation. To test this hypothesis, we studied two UV-C systems with the same specifications: a Spectroline HCL-1500 with BLE-1T155 15W 254 nm low-pressure amalgam bulbs (System 1), and a Spectroline Spectrolinker XL-1500 with third-party BLE-1T155 15W 254 nm low-pressure mercury bulbs (System 2) (Fig 5(b) of main text, and S15(a) Fig). Quantification of warm-up times (irradiance rise times), and output degradation over time are presented in S15(b) Fig. We observed distinct temporal irradiance variation as well as different maximum output (~11 mW/cm^2^ vs. ~7.7 mW/cm^2^), despite these systems having the same specifications. These irradiance variations propagate to the exposure time required to reach the critical UV-C dose. To extrapolate the effect these irradiance differences would have on N95 decontamination protocols, exposure times required to reach the marginally acceptable 1.0 J/cm^2^ dose at the center of the treatment plane were calculated for (i) the two near-identical systems, (ii) whether the bulbs are warmed-up prior to measurement, and (iii) whether the calculation is performed from measurements at the beginning or end of the exposure (S15(c) Fig). For System 1 after sufficient bulb warm-up, the average times required to deliver 1.0 J/cm^2^ dose only differed by 1.5% if calculated from the irradiance measured at the start and end of the exposure, indicative of relatively stable output over the treatment period. In contrast, the calculated average times to deliver the same dose in the same system without sufficient bulb warm-up differed by 29% (S15(c) Fig, top row), highlighting the importance of rigorously controlled bulb warm-up protocols. Following a similar trend, the calculated average times to deliver 1.0 J/cm^2^ without sufficient bulb warm-up for System 2 also differed by 29%. However, the absolute time required for System 2 to deliver 1.0 J/cm^2^ dose is far less than System 1 due to higher absolute irradiance. In contrast to System 1, the average times calculated to deliver 1.0 J/cm^2^ dose from the irradiance measured at the start and end of the exposure in System 2 after sufficient bulb warm-up still differed by 23% (S15(c) Fig, bottom row). This difference is due to an observed decline in irradiance over time, which we hypothesize is caused by the increased sensitivity to temperature of low-pressure mercury bulbs compared to low-pressure amalgam bulbs [3].

References:

1. Lawal O, Dussert B, Howarth C, Platzer K, Sasges M, Muller J, et al. Method for the Measurement of the Output of Monochromatic (254 nm) Low-Pressure UV Lamps. IUVA News. 19(1):9–16.

2. ASTM International. Standard Test Method for Determining Antimicrobial Efficacy of Ultraviolet Germicidal Irradiation against Influenza Virus on Fabric Carriers with Simulated Soil [Internet]. ASTM E3179 - 18. [cited 2020 Dec 3]. Available from: https://compass-astm-org.libproxy.berkeley.edu/download/E3179.35883.pdf

3. Lister GG, Lawler JE, Lapatovich WP, Godyak VA. The physics of discharge lamps. Rev Mod Phys. 2004 Jun;76(2):541–98.
